# Baseline household income is associated with severity and course of severe mental illness

**DOI:** 10.1017/S0033291726103341

**Published:** 2026-03-02

**Authors:** Juan Pablo Valencia-Arango, Juan Carlos Salazar-Uribe, Graciela Muniz-Terrera, Sara Wade, Danny Stevens Cardona, Johanna Valencia, Juan David Palacio-Ortiz, Ana María Diaz-Zuluaga, Jorge Vélez, Greta Gerdes, Marcelo Sanhueza-Vallejos, Robert McCutcheon, Kamaldeep Bhui, Philip McGuire, Loes Olde Loohuis, Nelson Freimer, Carlos López-Jaramillo, Nicolas A. Crossley

**Affiliations:** 1 SURA Colombia, Colombia; 2 Department of Statistics, Universidad Nacional de Colombia Sede Medellín, Colombia; 3 Ohio University Heritage College of Osteopathic Medicine, Ohio University, United States; 4 School of Mathematics, The University of Edinburgh, United Kingdom; 5 Department of Psychiatry, University of Antioquia, Colombia; 6 Center for Neurobehavioral Genetics, Semel Institute for Neuroscience and Human Behavior, UCLA: University of California Los Angeles, United States; 7 Centre for Research and Action on Social Determination and Mental Health (CIADES), Chile; 8 Department of Psychiatry, University of Oxford, UK; 9 Department of Psychiatry, Pontificia Universidad Católica de Chile, Chile

**Keywords:** bipolar disorder, multi morbidity, poverty, schizophrenia, severe mental illness, treatment resistance

## Abstract

**Background:**

Poverty is associated with the severity of common mental health disorders and increased physical comorbidities. However, its effects on severe mental illness (SMI), beyond increasing their incidence, are less understood, especially in low- and middle-income countries. We here examined the relationship between baseline household income and subsequent mental and physical health outcomes in a large cohort of individuals diagnosed with schizophrenia or bipolar disorder in Colombia.

**Methods:**

Retrospective cohort and case–control study using electronic health records from over 5 million Colombians. We identified individuals diagnosed with schizophrenia or bipolar disorder and their baseline household income. Mental health outcomes included third-line antipsychotic treatments (clozapine or antipsychotic polypharmacy) and psychiatric hospitalizations. Physical outcomes included diagnoses of hypertension, type 2 diabetes, and HbA1c levels, compared with rates in individuals without SMI.

**Results:**

We included 12,216 (6,485 women) participants newly diagnosed with bipolar disorder or schizophrenia between 2019 and 2023. Compared to middle-income participants (between $700–1,750USD/month), patients on a low income (less than $700USD/month) were more likely to require third-line antipsychotic treatment (OR 1.84 [1.64, 2.08]) and psychiatric hospitalization (incidence rate ratio 1.30 [1.21, 1.41]). Low-income participants with SMI had hypertension and diabetes rates like middle-income participants without SMI who were 20 years older. However, the combined effect of SMI and low income together posed a less-than-additive risk. Lower income was associated with higher HbA1c levels in diabetes, while a diagnosis of SMI was associated with lower levels.

**Conclusions:**

Low income at SMI onset is associated with worse mental and physical health outcomes.

## Introduction

A substantial body of evidence highlights the critical role that poverty plays in the development and clinical outcomes of common mental health disorders such as depression and anxiety (Ridley, Rao, Schilbach, & Patel, 2020). However, its impact on severe mental illness (SMI) such as schizophrenia or bipolar disorder, is less understood. Research on SMI has primarily focused on the bidirectional causal relationship: poverty increases the risk of developing SMI, and having a SMI leads to social decline and poverty (Crossley et al., 2022; Logeswaran, Dykxhoorn, Dalman, & Kirkbride, 2023; Marwaha & Johnson, 2004). The impact of living in poverty after individuals develop severe mental illness, and its associations with the subsequent course of the disorder and need for care have not been well examined (Jones et al., 2024). This knowledge gap may hinder the design and implementation of public policies that mitigate the effects of poverty on individuals with SMI (Mlay et al., 2022).

The influential World Health Organization International Study on Schizophrenia suggested that people with psychosis living in a poor environment had a better prognosis (Jablensky & Sartorius, 2008). Several authors have contested simplistic over-generalizations of these inter-country comparisons, such as ‘the ironic observation that abundance cripples’ (Cohen, Patel, Thara, & Gureje, 2008), and the associated notion that poverty might exert a lenient or even beneficial effect on severe mental illness (Burns, 2009). Such conclusions contradict evidence from high-income countries showing that low socioeconomic status is associated with higher risks of service disengagement (Phalen et al., 2024), poorer treatment response (Bennett & Rosenheck, 2021), and a lower probability of recovery (Peralta et al., 2022; Strakowski et al., 1998). Critically, there are only a few studies from low- and middle-income countries (LMIC), where individuals face the highest burden from economic hardship. For example, in Egypt, lower socioeconomic status among participants with bipolar disorder has been associated with longer delays in seeking treatment for mania(Ahmed, Elbeh, Khalifa, & Samaan, 2021). In China, low-income participants recently admitted for psychosis were more likely to be readmitted to hospital shortly after discharge (Ying et al., 2021).

Severe mental illness is also associated with multimorbidity (Halstead et al., 2024) and a reduced lifespan (Hayes et al., 2017). Social and economic inequalities have been shown to play a significant role on the disproportionate burden of non-communicable diseases on people with common mental health problems, not only by facilitating their coexistence but also by amplifying their interaction (Mendenhall et al., 2017). Multimorbidity poses a particular challenge for LMIC. These countries have a high burden of non-communicable disease and poorly developed health and social services, yet we have limited evidence of the scale and scope (Basto-Abreu et al., 2022).

To examine the role of poverty in shaping the clinical outcomes in severe mental illness and its potential effect on multimorbidity in a LMIC setting, we analyzed data from electronic health records of over 5.3 million people in Colombia. Our study focused on mental and physical health outcomes in relation to income among individuals newly diagnosed with bipolar or schizophrenia spectrum disorders between 2019 and 2023.

We hypothesized that patients with SMI and low income would present worse clinical outcomes (indicated by the need for third-line medication and the number of psychiatric admissions) compared to those with higher incomes. We also anticipated a higher risk of developing diabetes mellitus type 2 (DM2) or hypertension, along with a worse clinical status for DM2 (measured by the levels of glycated hemoglobin [HbA1c]), due to interactions with poverty. By quantifying the relationship between economic context and clinical outcomes in a LMIC setting, our findings may inform targeted interventions aimed at reducing disparities among vulnerable populations.

## Methods

### Study design and participants

The analyses reported include a retrospective cohort examining the impact of an exposure (poverty) on clinical outcomes in individuals with SMI, and a case–control study comparing individuals with and without SMI. A STROBE checklist can be found in the Supplementary Information.

We analyzed the electronic health records of individuals aged 18 to 60 years of age, diagnosed with schizophrenia spectrum disorder (F20-F29) or bipolar disorder (F30-F31) between 2019 and 2023, who were affiliated with SURA Health Insurance Company - EPS in Colombia. Colombia’s healthcare system provides universal coverage to its population, implemented through various healthcare providers, both private and public, known as ‘Entidades Promotoras de Salud (EPS)’. As of 2024, SURA-EPS was one of these providers, offering healthcare coverage to 5.3 million Colombians.

To be included in the analyses, participants needed to have information regarding the main household income and a follow-up of at least 12 months (i.e. a medical contact 12 months or more after the index consultation when the diagnosis of SMI was first made). As a control group, we included data from people affiliated with SURA-EPS within the same age range without a diagnosis of SMI. All data were anonymized before being analyzed by the research team.

### Income and other characteristics of participants

Income was obtained from the records of the insured individual and their dependents. Participants were categorized into three income groups based on their individual earnings or family members that they depended on. These groups are defined in the Colombian system regulating income-adjusted contributions for healthcare costs, where higher earners and their dependents contribute more:

- low income: under 2 minimum salaries.
- middle income: between 2 and 5 minimum salaries.
- high income: more than 5 minimum salaries.

Colombia’s current minimum salary is 350USD per month.

Sex was obtained from the insurance records that correspond to the sex assigned at birth of participants. Information about race, household size, and marital status are seldom asked and recorded in the system in this context, with many missing data, and therefore are not included in the analysis.

### Clinical outcomes

To assess the impact of poverty on clinical outcomes in SMI, we looked at markers of severity available on the electronic health records, specifically:

- time to initiation of third-line medication treatment since diagnosis, specifically time to initiation of clozapine or more than two antipsychotics.
- number of admissions to a psychiatric hospital.

To explore the relation between poverty, psychosis and cardiometabolic disorders, we examined:

- percentage diagnosed with diabetes mellitus type 2 (DM2, ICD-code E11).
- percentage diagnosed with essential hypertension (ICD-10 code I10).

Finally, to examine level of care of patients with DM2, poverty, and SMI, we explored HbA1c levels when available.

Sensitivity analyses explored these outcomes according to diagnosis (schizophrenia or bipolar disorder).

### Statistical analyses

We used generalized linear models to examine the different outcomes, controlling for age and sex, and using the middle-income group as reference.

For our retrospective cohort examining the impact of income on clinical outcomes, we conducted:

- Kaplan–Meier analyses with a log-rank test to compare survival times examining time to initiation of third-line treatment and its relationship with income.
- Logistic regression examining the risk of initiating third-line treatment medications at 2 years between different income groups of the general form:

(1)

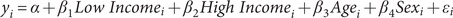

- For number of psychiatric admissions, we used a Poisson regression model with a similar form as Equation 1 above, including a dummy variable flagging admissions at years 3 or 4 after diagnosis and their interaction with income to account for temporal trends in psychiatric admissions.
- For our case–control analyses looking at SMI, income, and metabolic disorders, we used logistic regression models of the general form:

(2)

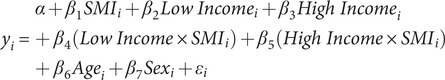

- For the HbA1c analysis, we included time since DM2 diagnosis as a covariate and applied a mixed linear model to examine HbA1c levels, incorporating a random factor (*u_i_*) to account for repeated measures in some participants:

(3)

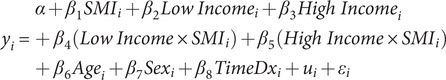



All analyses were done using R Statistical Software (R Core Team, 2018).

### Ethical approval

This study was approved by the Ethics Committee ‘Comité de ética y BPC en investigación en salud’ from SURA Health Insurance (Record Nr 127–13/03/2024). Consent was not requested from individual participants as approved by the committee as data were anonymized. The authors assert that all procedures contributing to this work comply with the ethical standards of the relevant national and institutional committees on human experimentation and with the Helsinki Declaration of 1975, as revised in 2008.

## Results

### Characteristics of the sample

**Figure 1.**
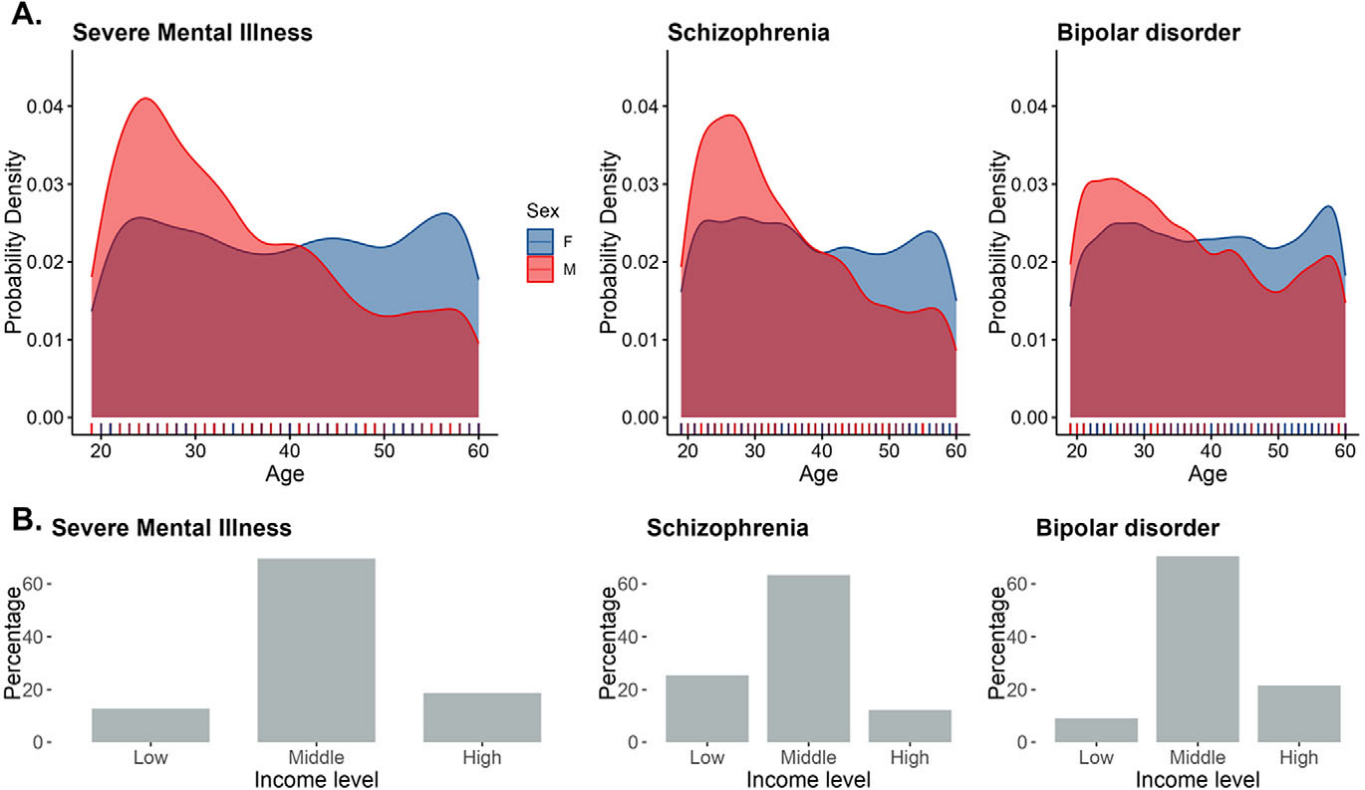
Characteristics of the sample of 12,216 participants included. (a) Age distribution of men and women at the time of first diagnosis. (b) Proportion of participants across different income levels.

From a total population of over 5.3 million insured individuals, data were available from 12,216 people aged 18 to 60 years old (6,485 women), who had received a first diagnosis of severe mental illness (SMI; either bipolar disorder or schizophrenia) between 2019 and 2023. Figure 1 provides demographic and clinical information about the participants. Individuals might have been diagnosed with either disorder at different assessment points during their follow-up period: 1,690 individuals were diagnosed only with schizophrenia spectrum disorders, 6,916 only with bipolar disorder, and 3,610 received both diagnoses (Supplementary Figure S1). The follow-up duration (time from diagnosis to the last available mental health contact) was 26 months (interquartile range: 18 to 40 months).

Information about income was present for all patients in the insurance database, since this is used to calculate the costs of their healthcare out-of-pocket costs. However, 3,492 patients received a diagnosis of severe mental illness in a mental health consultation but had no further contact with any of the health services in the follow-up period and were therefore not included in the analysis. Comparisons of the demographic characteristics of this group with the included participants are shown in Supplementary Table S1. Excluded participants were more likely to be women, of higher-income groups, and with a diagnosis of schizophrenia. Flowcharts describing the number of eligible participants included and excluded across the different analyses are shown in Supplementary Figures S2 (for severe mental illness) and S3 (separately for schizophrenia and bipolar disorder).

### Effect of baseline income on clinical outcomes in SMI

**Figure 2.**
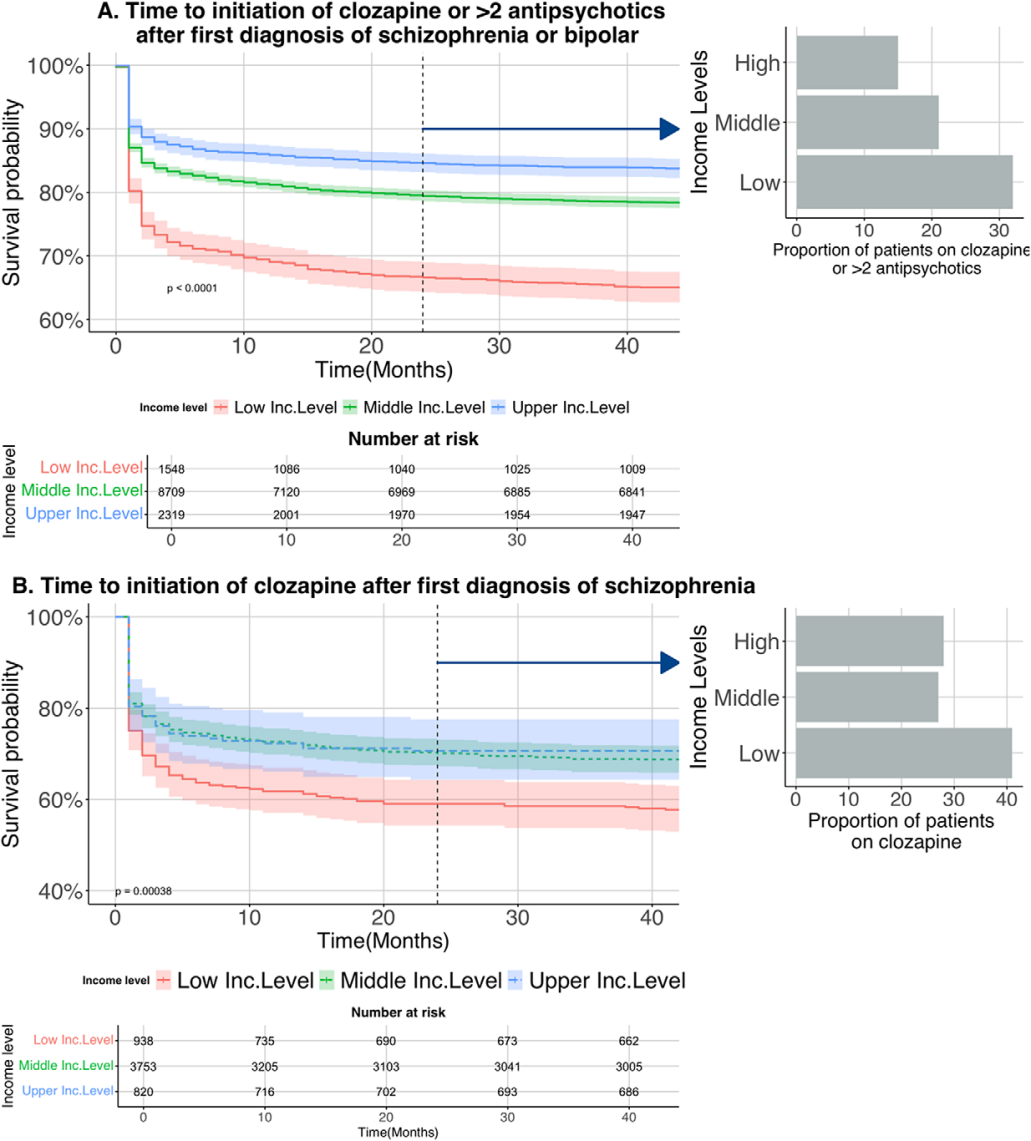
Time to initiation of third line treatment in psychosis. (a) Survival time for people diagnosed with a psychotic disorder before being started on > 2 antipsychotics or clozapine. Inset shows the risk for the different incomes at 2 years follow-up. (b) Probability of clozapine prescription in people diagnosed with schizophrenia.

There was a clear income-gradient on time to initiation of clozapine or more than two antipsychotics in adults diagnosed with SMI (Figure 2a; log-rank test, *P* < 0.001). Two years after diagnosis, people in the low-income group were 84% more likely to have started third-line antipsychotic treatment compared to those on a middle income (OR 1.84 [1.64, 2.08]; see Supplementary Table S2A for full model results). Those in the higher-income group were less likely to be prescribed these medications compared to those on a middle income (OR 0.72 [0.64, 0.82]). Because our bipolar disorder sample included individuals without psychotic symptoms, in whom clozapine is less commonly used, we also examined clozapine use in the subgroup of individuals diagnosed with schizophrenia (Figure 2b, Supplementary Table S2B). Again, those with a diagnosis of schizophrenia and on low income were 53% more likely to have started clozapine by 24 months (OR 1.53 [1.22, 1.93]) than those on middle income. There was no evidence of a differential effect related to subgroup of disorder or outcome (Supplementary Figure S4).

**Figure 3.**
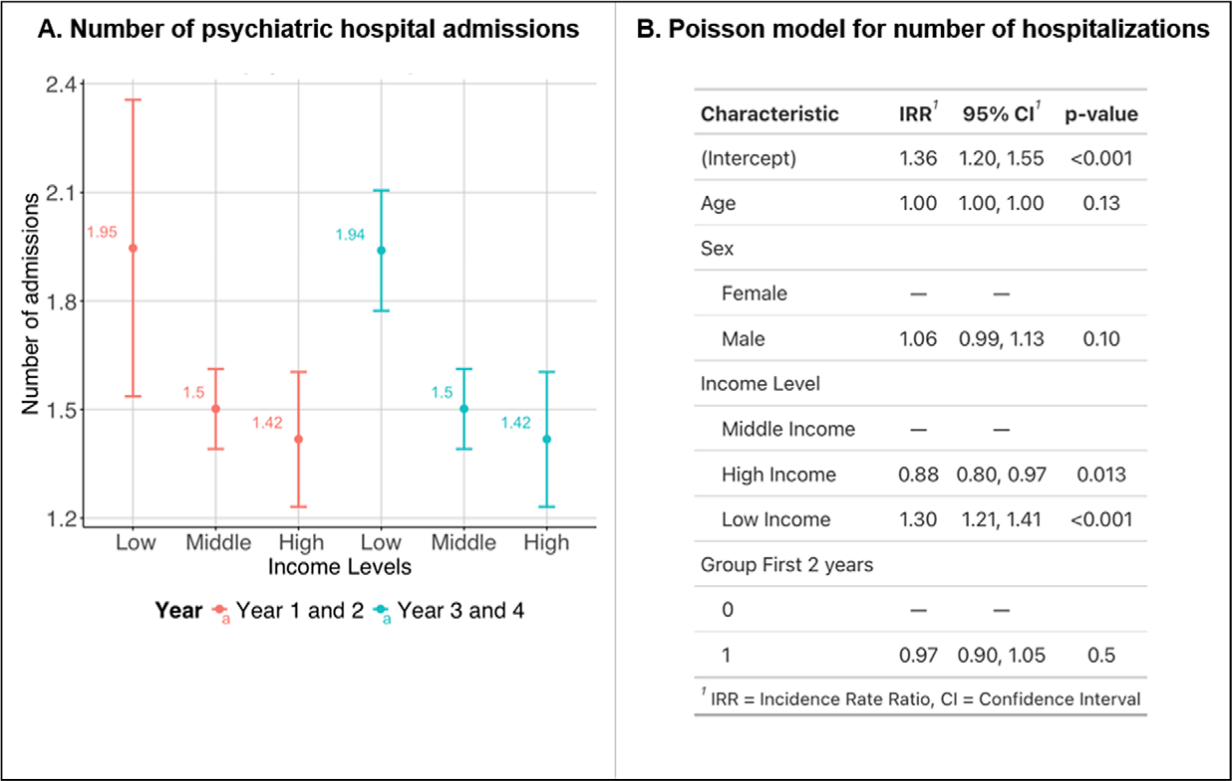
Psychiatric admissions according to income level. (a) Mean number of admissions and 95% confidence interval for the first 2 years (red) and second 2 years (green). (b) Poisson model for number of hospitalizations by patient.

A similar socioeconomic gradient emerged in relation to the number of psychiatric hospital admissions. As shown in Figure 3, among the total SMI sample, patients on low income had 30% higher number of hospital admissions compared to those on middle incomes (incidence rate ratio [IRR] 1.30 [1.21,1.41]). Conversely, those with higher incomes had less admissions than those with middle incomes (IRR 0.88 [0.80, 0.97]). The higher admission rates in patients with low compared to middle incomes was driven by participants with a diagnosis of schizophrenia (Supplementary Table S3). There was no significant difference between admissions in the first 2 years compared to the following third and fourth year.

### Income gradient and the comorbid presentation of SMI and diabetes or hypertension

To examine the prevalence of comorbid hypertension and type 2 diabetes mellitus (DM2) with SMI, we compared our cohort with 3,737,870 adults without a history of SMI.

**Figure 4.**
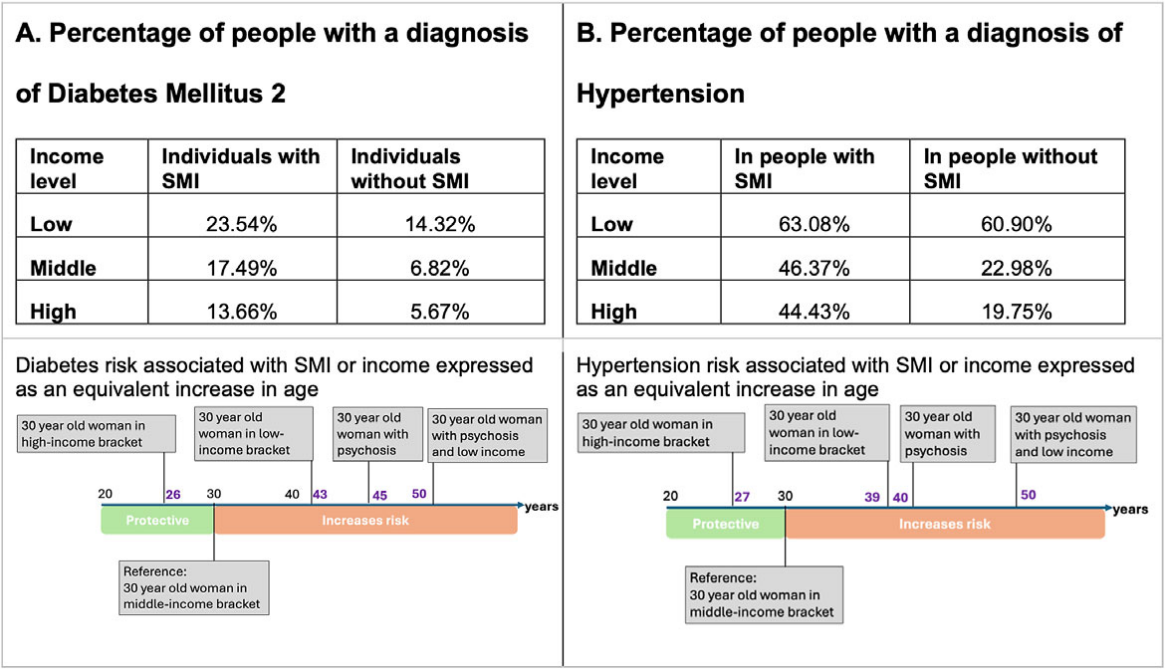
Risk of diabetes and hypertension related to SMI. Unadjusted rates and equivalent risk expressed in ages from models examining the relationship between SMIs and diabetes mellitus 2 (a) and hypertension (b). We calculated the equivalent risk for four different scenarios using as a reference a 30 year old woman without SMI in the middle-income bracket: a 30-year-old woman in the high-income bracket, 30-year-old woman in the low-income bracket, a 30-year-old woman with SMI and middle-income, and a 30-year-old woman with SMI and low income.

Unadjusted rates of DM2 in different groups are reported in Figure 4a. After adjusting for age and sex, individuals diagnosed with SMI had an odds ratio of being diagnosed with DM2 of 3.37 [3.09, 3.68]. Being in the lower income bracket, compared to the middle-income bracket, was associated with an increased odds of diabetes of 2.99 [2.94, 3.03]; being in the highest income bracket was linked to a 26% further reduction (odds ratio of 0.745 [0.74–0.75]). Finally, we found a significant negative interaction between having a diagnosis of SMI and being in the lowest income bracket (compared to the middle), with the odds ratio modified by 0.54 [0.42–0.69], suggesting that the increased risk associated with poverty and SMI were not additive. Details of the model are presented in Supplementary Table S4A. Models for individuals with and without SMI exploring the association with income and diabetes prevalence are shown in Supplementary Table S5 to help grasp the negative interaction between SMI and income level.

To better understand the magnitude of these effect sizes, we compared them to the risk conferred by ageing (Figure 4a, see Supplementary Information for a more detailed explanation of the method use). A 30-year-old person diagnosed with SMI on a middle income would have the same risk of developing diabetes as a 45-year-old person with the same income level but no SMI. A 30-year-old person from the lowest income group would have the same risk as a person on a middle income at age 43, while a 30-year-old person in the highest income bracket would have the same risk as a 26-year-old on a middle income. A person with both SMI and low income would have the same risk as someone without SMI and on middle income at age 50.

A similar pattern is evident for the risk of hypertension, as shown in Figure 4b (unadjusted rates) and Supplementary Table S4B (model results). Psychosis and poverty were both linked to higher odds of hypertension, with odds ratio for SMI of 3.03 [2.82, 3.26] and for poverty 2.63 [2.59, 2.66]). A higher income was associated with lower odds (a decrease in odds ratio of 0.72 [0.72, 0.73]). Typically, a higher income corresponded to reduced odds of hypertension, yet in cases where individuals had also SMI, this association was less pronounced (OR 1.26 [1.08, 1.48]). No significant interaction was observed between a diagnosis of SMI and low income for hypertension. Effect sizes were large, with people with SMI and on low income having the equivalent risk of a person 20 years older who does not have SMI and is in the middle-income bracket (Figure 4b).

Results remained substantially unchanged when looking at schizophrenia or bipolar disorder separately (Supplementary Tables S6 and S7).

## Diabetes control in people with SMI and different incomes

We finally analyzed HbA1c levels for 528 patients with a diagnosis of DM2 and SMI (median age 52 [IQR 45,57]; 63% women; median number of HbA1c measurements = 4), compared to 121,293 patients with DM2 without SMI that had measurements (median age 61 [52,70]; 56% women; median number of HbA1c measurements = 6). A mixed-model analysis including all observations found that, contrary to our initial hypothesis, people with DM2 and SMI had lower HbA1c compared to those with DM2 and without SMI (−0.18 average points lower [−0.03, −0.33]; Supplementary Table S8). This effect was driven by better HbA1c levels in individuals diagnosed with bipolar disorder across income groups, and those with schizophrenia who were on a low income (Supplementary Table S9). Household income had an independent effect, with individuals on low income having significantly higher HbA1c (0.33 points higher [0.29, 0.37]), while those with higher income had significantly lower values (−0.13 points lower, [−0.12, −0.15]), than those on a middle income. No significant interaction between SMI and income levels in HbA1c was observed in people with DM2.

## Discussion

Individuals who develop SMI face a range of complex adversities that can lead to poor outcomes across multiple dimensions (Jones et al., 2024). Our data highlight that household income when people became unwell with SMI is strongly associated with both mental and physical health outcomes. People with SMI on a low household income were likely to initiate third-line antipsychotic treatment, be admitted to psychiatric hospitals, and develop hypertension or DM2.

Our results are noteworthy as they quantify the strong effect associated with clinical outcomes and low income across mental health indicators in a LMIC setting. People with SMI and with low incomes were approximately 30% and 40% more likely to be admitted to hospital than those with middle and high incomes, respectively. These findings are in line with the socioeconomic gradient reported in admissions for psychosis in high-income countries (Suokas et al., 2020), echo findings on 6-month readmission rates in China (Ying et al., 2021), and recent data from our group from a different region in Colombia (Gerdes et al, 2025). In this study, people with schizophrenia who had a low income were 53% more likely to develop treatment resistance (as indexed by treatment with clozapine) within 2 years of diagnosis compared to those with middle incomes. There have been fewer studies addressing this, with studies relating treatment resistance to educational attainment (Wimberley et al., 2016).

We also found that low income was strongly associated with physical comorbidity. A person with SMI and low income at age 30 had the equivalent risk of developing DM2 or hypertension of someone 20 years older without SMI and in the middle-income bracket. It is highly likely that there is also a strong socioeconomic gradient in the shorter lifespan associated with SMI (Hayes et al., 2017).

Income poverty is a potentially actionable target in SMI, but has been relatively neglected in research, with a few notable exceptions (Mlay et al., 2022; Tsai, McCleery, Wynn, & Green, 2023). The mechanisms through which poverty exert its impact on SMI are likely to be multifactorial. One potential factor is a lack of access to healthcare. However, in this study, the participants were managed by Colombia’s EPS system, which provides comprehensive healthcare access to all those insured, suggesting that additional factors are playing a role. Our work in Colombia proposes that challenges in healthcare access associated with poverty are also related to geographical distance (Song et al., 2024). Moreover, it is acknowledged that poverty can influence health outcomes through mechanisms such as worse early life conditions, limited education, inadequate nutrition, exposure to pollution, unemployment, and violence and crime (Ridley et al., 2020). There is an opportunity to learn from the development of interventions targeting poverty in common mental health disorders such as direct or conditional cash transfers (Evans-Lacko et al., 2023). Furthermore, poverty is a multidimensional state of disadvantage including dimensions such as absolute poverty, relative poverty and perceived inequality, or financial stress. To design effective interventions, there is a need to identify specific aspects of deprivation driving these associations.

Contrary to our initial hypothesis, we did not observe an interaction effect between different types of adversities. Thus, the combination of poverty plus SMI did not increase the risk of comorbid DM2 or hypertension beyond the additive risks of both, and in several cases, it was slightly lower. The effect sizes associated with both SMI and low income in the Colombian context were already very large, which may have made it difficult for the risk to increase beyond their additive effect (ceiling effect). A similar absence of interaction was seen in a study that found lower relative risks (but not absolute) of stroke and ischemic heart disease associated with schizophrenia in deprived areas in Scotland compared to those from more affluent areas (Jackson et al., 2020). Possibly SMI and income share mechanisms leading to a higher risk of DM2 or hypertension. For example, SMI is associated with increased sedentary behavior (Stubbs et al., 2016), while higher income is linked with increased leisure physical activity (Gidlow et al., 2006). The existence of shared mechanisms also implies that interventions are most likely to benefit individuals if they target the multiple adversities that they face.

Diabetes care, in terms of levels of HbA1c achieved, was better for people with SMI, both for bipolar disorder and those on a low income and with a diagnosis of schizophrenia. The existing literature looking at diabetic care and SMI has reported mixed results: some studies indicated a negative effect of SMI, while others reporting similar or even improved care (Frayne et al., 2005; Knudsen et al., 2023). Overall, our findings suggest positive developments in healthcare for vulnerable individuals with severe mental illness, while underscoring the importance of comprehensive care packages that extend beyond healthcare provision.

Strengths of this study include the use of data from a LMIC country such as Colombia, populations that are under-represented in the SMI literature, particularly in studies involving electronic health records. Moreover, we had access to both medical and psychiatric consultations, which are often handled by separate institutions in other countries. Colombia’s EPS system provides comprehensive healthcare access to those insured. However, access to certain psychosocial interventions may not be fully covered and could have been more readily available to participants who could afford to pay for them independently. On the other hand, income groups were based on the earnings of the insured individual or their insured family members if they were dependent. We did not correct this income for household size or considered other sources of family income, which would have provided a more accurate picture of the financial support of the patient. A total of 3,492 potential participants diagnosed with severe mental illness were not included due to a lack of follow-up data. These individuals were similar in age but included a higher proportion of women. They were more likely to belong to higher-income groups, who may have accessed mental health support privately outside the insurance system. They were also more likely to have received a diagnosis of schizophrenia. This comparison does not consider all included participants who received a diagnosis of both bipolar and schizophrenia at different consultations, which may have artificially inflated the number of schizophrenia patients appearing to be lost to follow-up. Finally, our study relied on diagnoses extracted from clinical health records, which were not based on structured diagnostic assessments. This may partly explain the large number of participants who received both diagnoses of bipolar and schizophrenia. However, diagnostic changes over time are not uncommon after a first episode of psychosis, even when applying more rigorous diagnostic assessments. Studies have shown that diagnoses change in approximately 10% of those initially diagnosed with schizophrenia, 16% of those with affective psychosis spectrum, and up to 28% of those with schizoaffective disorder (Fusar-Poli et al., 2016).

In summary, we conclude that poverty is strongly linked to a worse clinical course of schizophrenia and bipolar disorder in a LMIC setting, as well as to their associated risk of multimorbidity. This is an actionable target on a targeted group of people living with these disorders, with a potential significant impact on their lives.

## Supporting information

10.1017/S0033291726103341.sm001 Valencia-Arango et al. supplementary material Valencia-Arango et al. supplementary material

## Data Availability

Individual-level data from this study are not publicly available. However, the authors are open to collaborative research and can provide aggregated data or conduct joint analyses upon reasonable request. Interested researchers are encouraged to contact the corresponding authors for further information. Analytic codes used are available upon requests.

## Supplementary material

The supplementary material for this article can be found at http://doi.org/10.1017/S0033291726103341.
