# Supplementary material for: Baseline household income is associated with severity and course of severe mental illness
Source: Psychol Med. 2026 Mar 2;56:e59. doi: 10.1017/S0033291726103341 (PMC12969196; doi:10.1017/S0033291726103341)
Supplement: Valencia-Arango et al. supplementary material [file S0033291726103341sup001.docx]

***Supplementary Information for:***

**Baseline household income is associated with severity and course of severe mental illness.**

JP Valencia-Arango, JC Salazar-Uribe, G Muniz-Terrera, S Wade, DS Cardona, J Valencia, JD Palacios, AM Diaz, J Vélez, G Gerdes, M Sanhueza, R McCutcheon, K Bhui, P McGuire, L Olde Loohuis, N Freimer, C López-Jaramillo, NA Crossley.

*Methods*

Method to represent risk based on age (Figure 4)

*Tables*

- S1 – Characteristics of participants with missing data.
- S2 – Associations between household income level and subsequent treatment.
- S3 – Number of admissions and income levels in schizophrenia and bipolar disorder.
- S4 - Risk of diabetes and hypertension related to a diagnosis of SMI.
- S5 – Risk of diabetes according to income level in participants with a diagnosis of SMI and (separately) without.
- S6 - Risk of diabetes and hypertension related to a diagnosis of schizophrenia.
- S7 - Risk of diabetes and hypertension related to a diagnosis of bipolar disorder.
- S8 - HbA1c levels in those with DM2, and with or without a severe mental illness diagnosis.
- S9 - HbA1c levels in those with DM2, and with or without a diagnosis of schizophrenia or bipolar disorder.

*Figures*

- S1 – Frequency in which individuals received a diagnosis of schizophrenia spectrum disorder or bipolar disorder during their different clinical encounters.
- S2 – Flowchart of eligible participants with SMI included and excluded across the different analyses.
- S3 – Flowchart of eligible participants with schizophrenia and bipolar disorder included and excluded across the different analyses.
- S4 – Survival curves for probability of starting third-line antipsychotic treatment across subgroups and outcomes.

*STROBE checklist*

**Supplementary Methods**

**Method to represent risk based on age (Figure 4)**

To interpret the estimated risk of Diabetes or Hypertension of a specific case profile in terms of age, we start from our general linear model:

$$logit({P(y}_{i}=1)=\alpha+\beta_{1}{{SMI}_{i}+\beta}_{2}{Low Income}_{i}+\beta_{3}{High Income}_{i}+\beta_{4}{(Low Income\times SMI}_{i})+\beta_{5}{(High Income\times SMI}_{i})+\beta_{6}{Age}_{i}+\beta_{7}{Sex}_{i}+\varepsilon_{i}$$

For the reference profile of being a woman on middle income without SMI (all reference) and aged 30, then the linear predictor (log-odds) is

$$\eta_{ref}= \alpha+\beta_{6}\times30$$

Accordingly, for a female of similar age on low income, the linear predictor is

$$\eta_{lowinc}= \alpha+\beta_{2}+\beta_{6}\times30$$

So, to find the age *A* such that a middle-income woman would have the same risk (same log-odds) as the woman on low income we solve:

$$\alpha+\beta_{6}\times A= \alpha+\beta_{2}+\beta_{6}\times30$$

$$A= 30+\frac{\beta_{2}}{\beta_{6}}$$

Similarly, a low-income woman with SMI at age 30 has the same predicted risk as our reference person (middle-income woman without SMI) aged *A_2_*:

$$A_{2}= 30+\frac{\beta_{1}+\beta_{2}+\beta_{4}}{\beta_{6}}$$

**Supplementary Tables**

**Table S1. Characteristics of participants with missing data**

|  | | **Included** | **Excluded** | **Comparison** |
| --- | --- | --- | --- | --- |
| **N** | | 12,216 | 3,492 |  |
| Gender (% female) | | 6,485 (53.1%) | 2,251 (64.4%) | Chi-squared = 141.9,  df = 1, *P* < 0.0001 |
| Age (years – SD) | | 38.8 (12.7) | 38.6 (12.6) | t-stat = 0.826, *P* = 0.4 |
| Income | Low | 1,506 (12.3%) | 205 (5.9%)* | Chi-squared = 181.7, |
|  | Middle | 8,458 (69.2%) | 2380 (68.2%) | df = 2, *P* < 0.0001 |
|  | High | 2,252 (18.4%) | 907 (26.0%)* | * post-hoc *P* < 0.05 |
| N Bipolar (%) | | Only – 6,916 (80.4%) | 2,510 (71.9%) | Chi-squared = 103.4, |
| N Schizophrenia (%) | | Only – 1,690 (19.6%) | 982 (28.1%) | df = 1, *P* < 0.0001 |

SD = standard deviation, df = degrees of freedom.

Note that comparisons between proportion of diagnoses of bipolar disorder and schizophrenia in the included group only refer to participants whose diagnosis was consistent during the follow-up period.

**Table S2. Associations between household income level and subsequent treatment.**

| A. Full model examining associations between income level and third-line medication treatment at 2 years. | B. Full model examining associations between income level and clozapine prescription in schizophrenia at 2 years. |
| --- | --- |
| 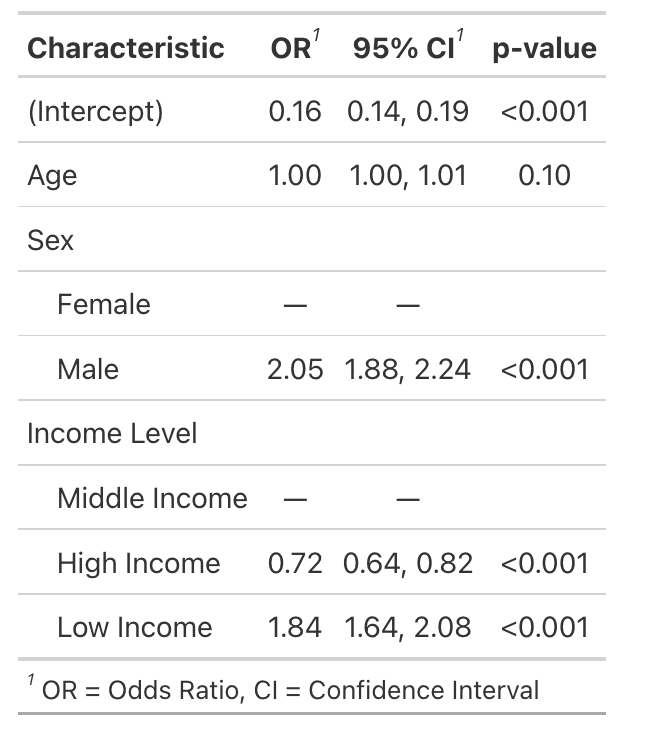 | 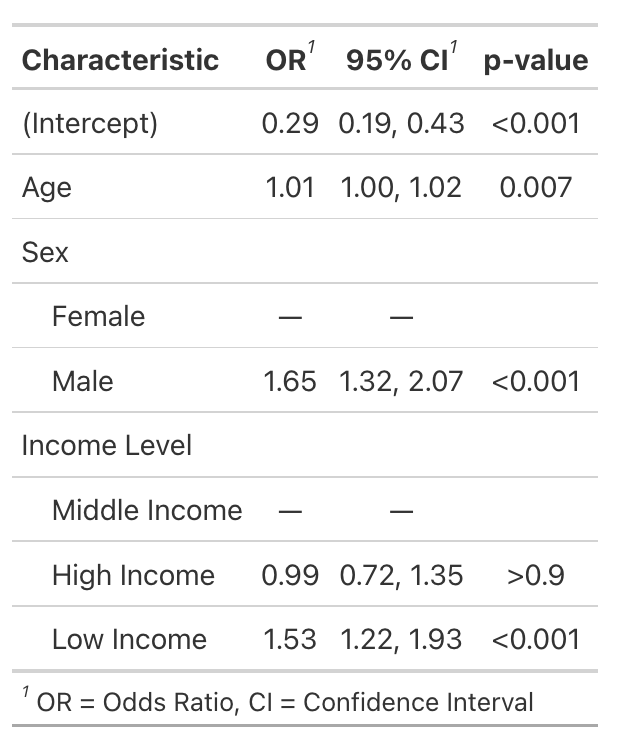 |

**Table S3. Household income and admission rates in schizophrenia and bipolar disorder.**

| A. Full model examining associations between income level and number of admissions in patients with schizophrenia.  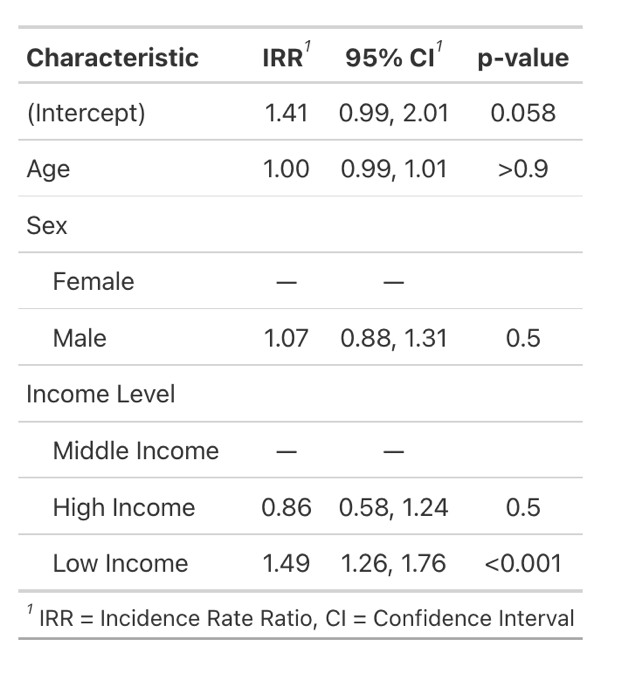 | B. Full model examining associations between income level and number of admissions in patients with bipolar disorder.  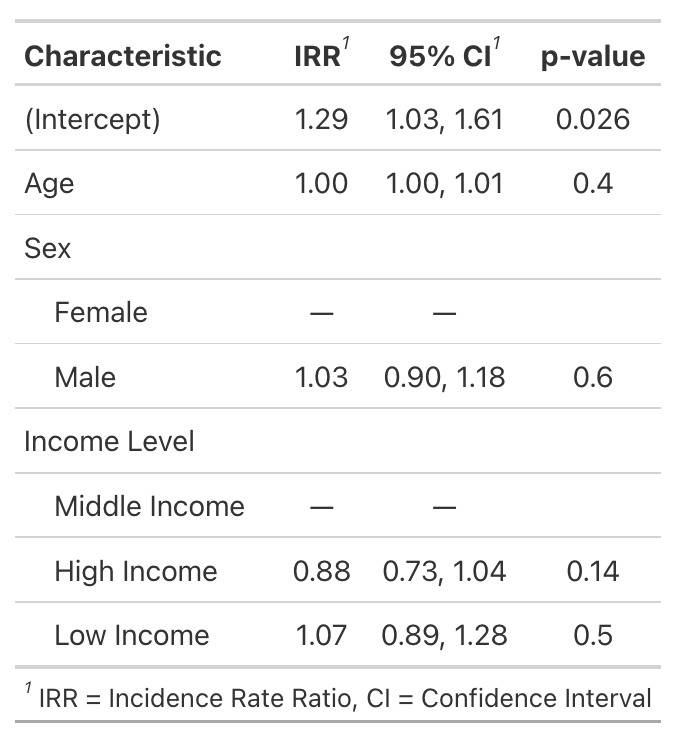 |
| --- | --- |

**Table S4. Risk of diabetes and hypertension related to a diagnosis of severe mental illness.**

| **A- Risk of Diabetes Mellitus 2**  **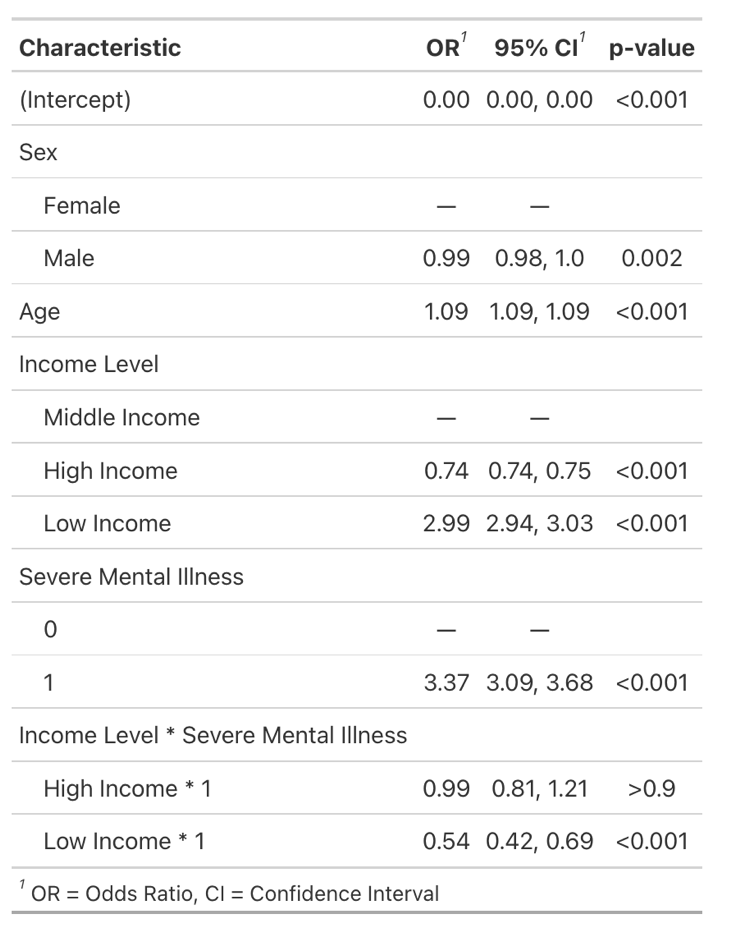** | **B- Risk of Hypertension**  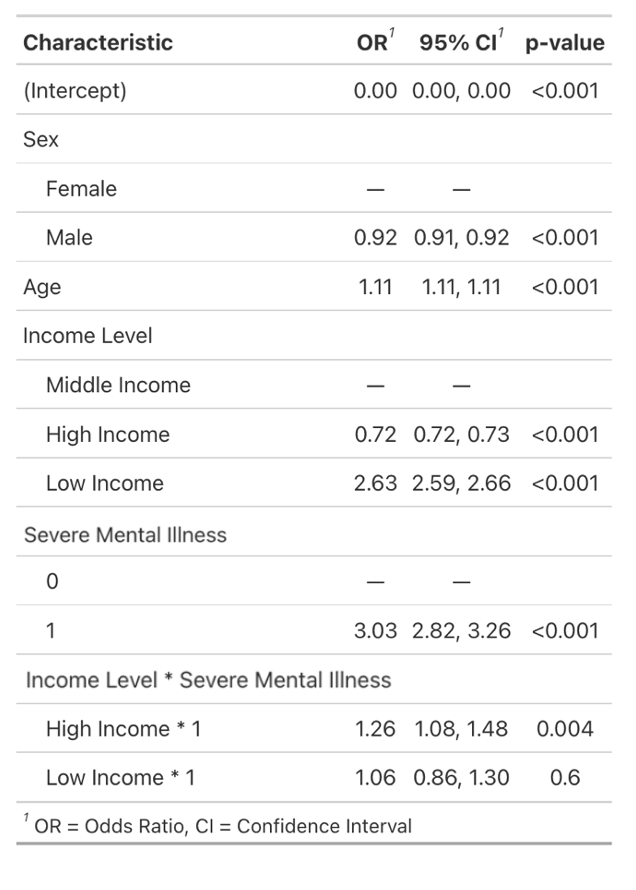 |
| --- | --- |

**Table S5. Risk of diabetes according to income level in participants with a diagnosis of SMI and (separately) without.**

| 1. **Patients with SMI**   **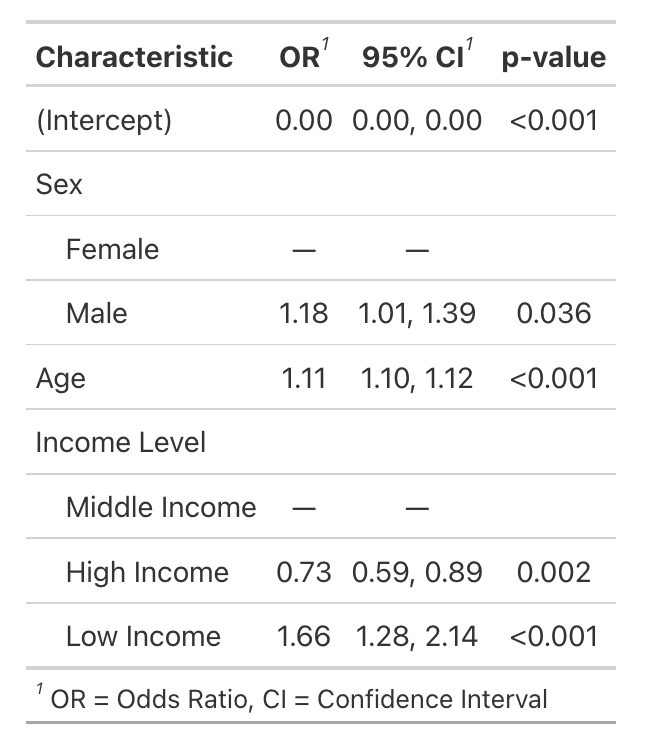** | 1. **Patients without SMI**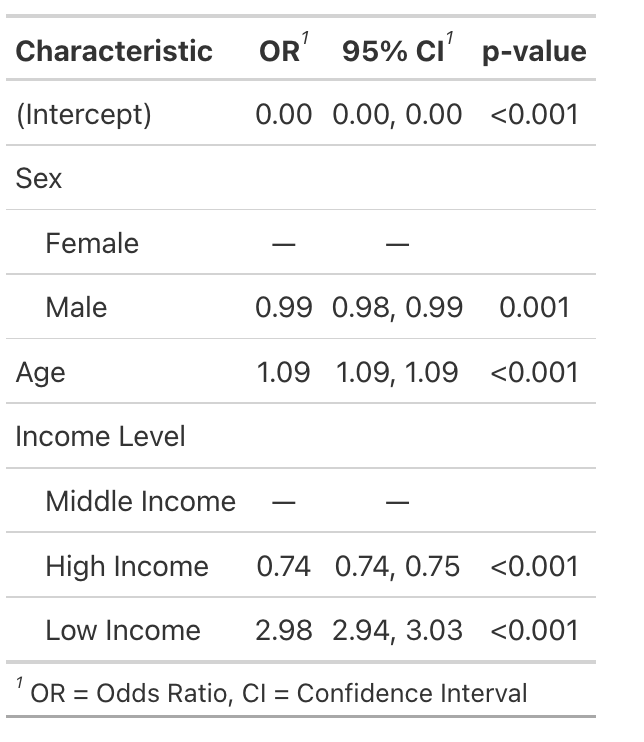 |
| --- | --- |

**Table S6. Risk of diabetes and hypertension related to a diagnosis of schizophrenia.**

| **A- Risk of DM2 and Schizophrenia**  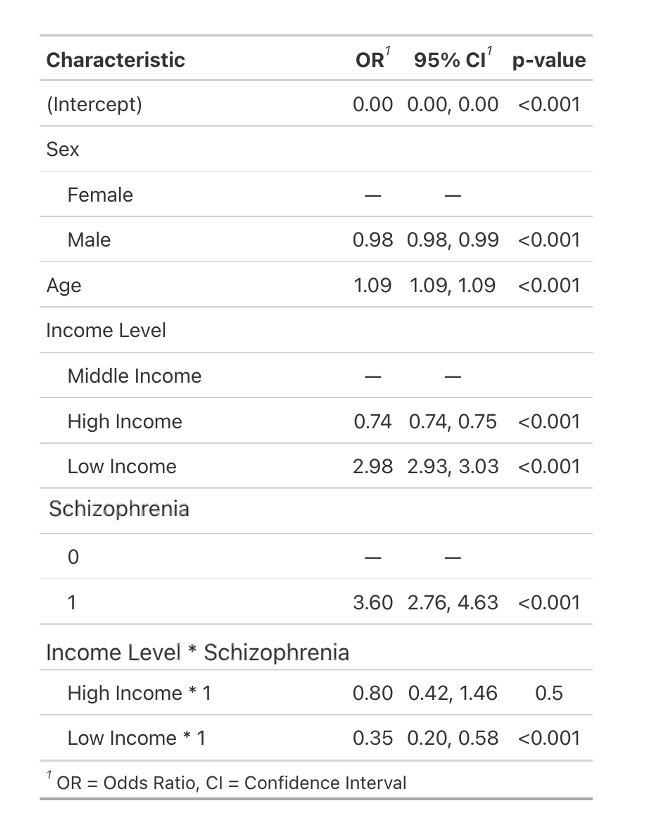 | **B- Risk of Hypertension and Schizophrenia**  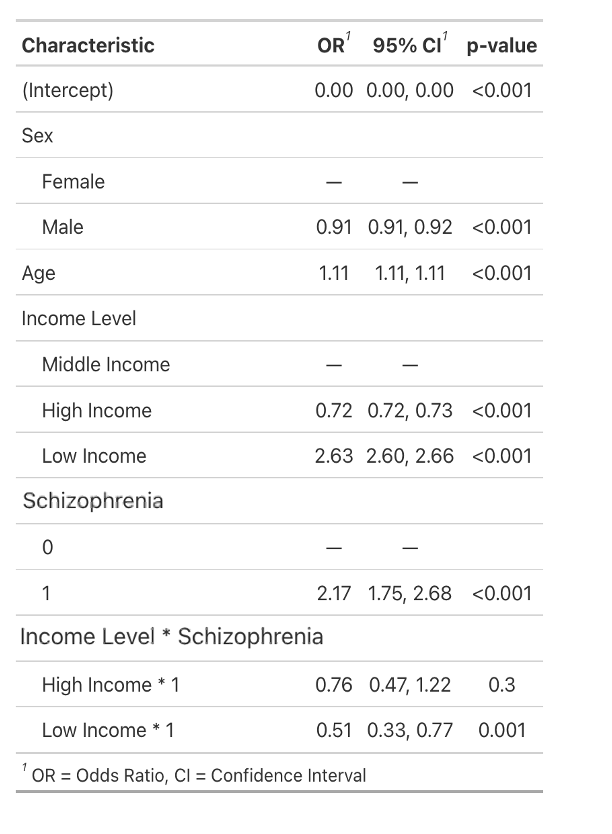 |
| --- | --- |

**Table S7. Risk of Diabetes and Hypertension in Bipolar Disorder according to income level.**

| 1. **Risk of DM 2 and Bipolar Disorder**   **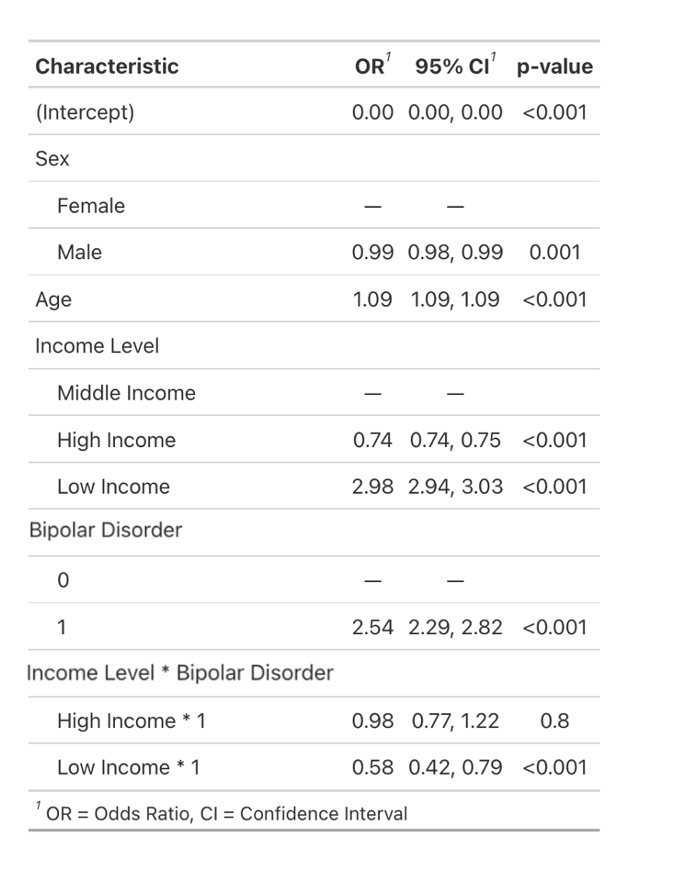** | **B- Risk of Hypertension and Bipolar Disorder**  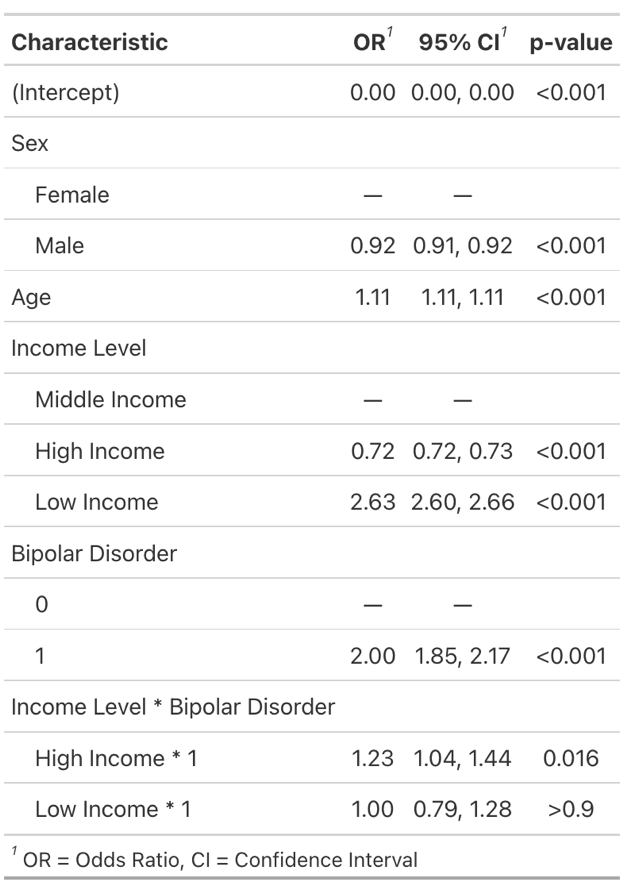 |
| --- | --- |

**Table S8*.* HbA1c levels in people with DM2 and severe mental illness.**

| A. HbA1c levels in those with DM2, and with or without a diagnosis of severe mental illness.   |  |
| --- | --- |

**Table S9*.* HbA1c levels in people with DM2 and schizophrenia or bipolar disorder.**

| A. HbA1c levels in those with DM2, and with or without a diagnosis of schizophrenia  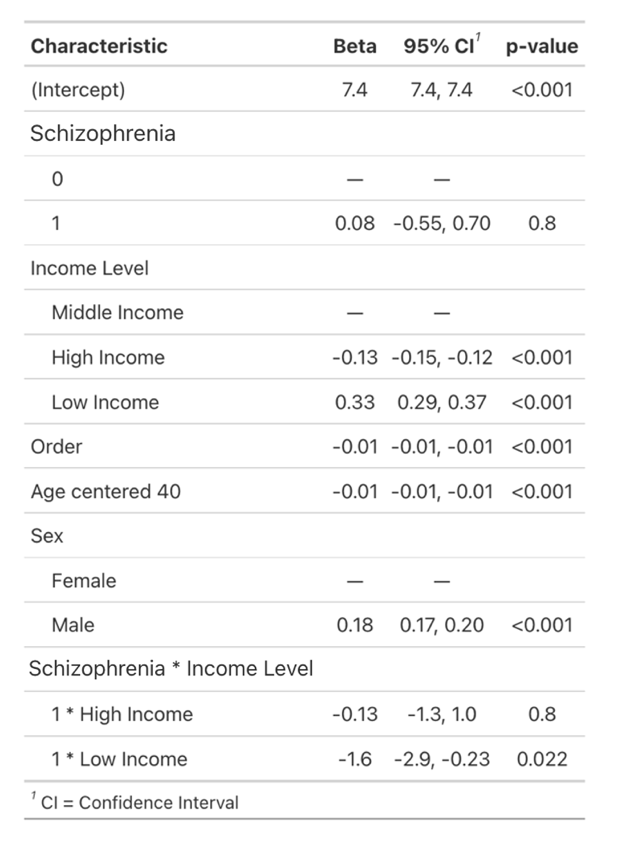 | B. HbA1c levels in those with DM2, and with or without a diagnosis of bipolar disorder  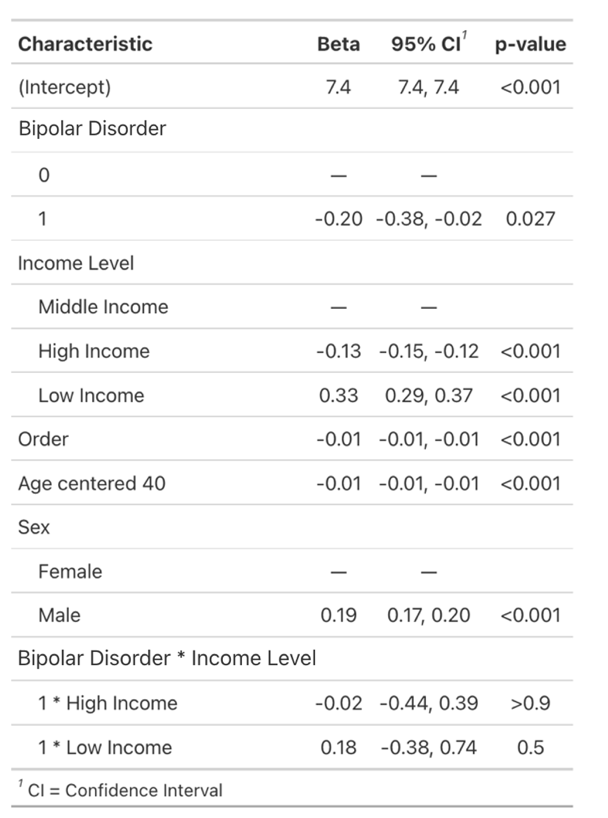 |
| --- | --- |

**Supplementary Figures**

**Figure S1**


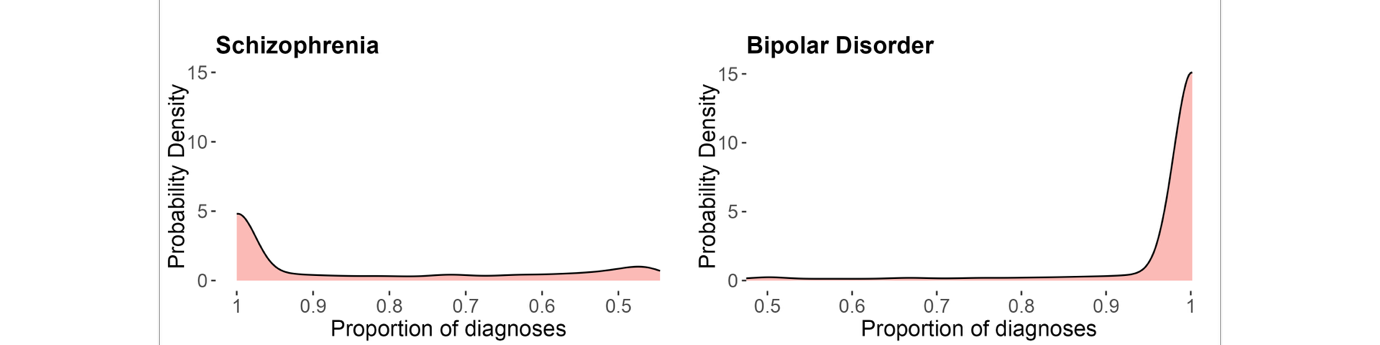


***Figure S1. Frequency in which individuals received a diagnosis of schizophrenia or bipolar disorder in different consultations.*** *The distributions are clearly bimodal, indicating that even those who received both diagnoses during the study period, there was a predominant diagnosis, either schizophrenia or bipolar disorder.*

**Figure S2**

***Figure S2. Flowchart of eligible participants with SMI included and excluded across the different analyses.***

**Figure S3**

***Figure S3. Flowchart of eligible participants with schizophrenia or bipolar disorder included and excluded across the different analyses.*** *Note that combined number of participants is larger than the total included in the pooled severe mental illness analysis, as some individuals received both diagnoses at different time points and were counted in each relevant category.*

**Figure S4**

*
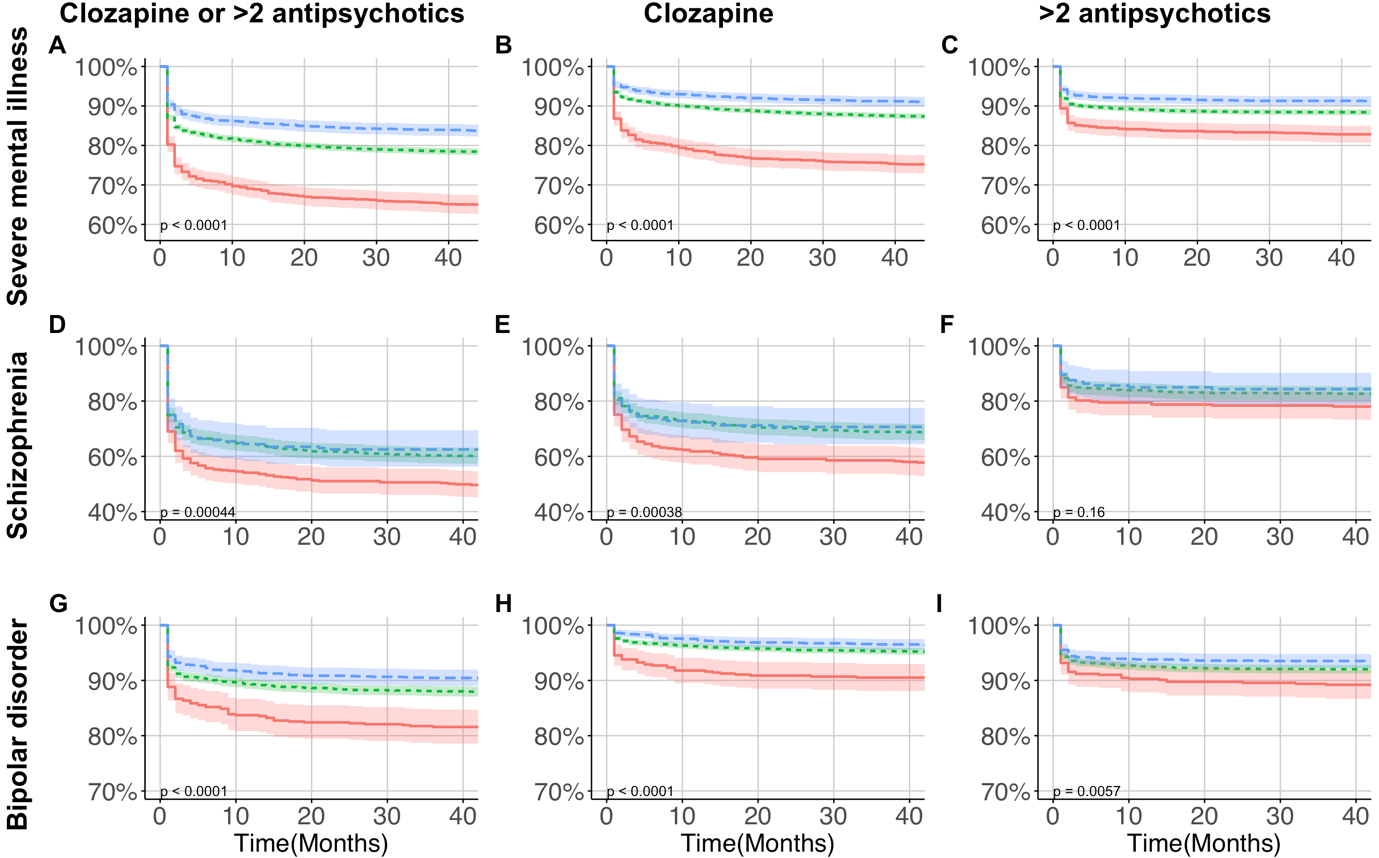
*

***Figure S4. Survival curves showing time to initiation of clozapine or more than two antipsychotics in severe mental illness according to income.*** *95% confidence intervals shown.*

# STROBE Statement—checklist of items that should be included in reports of observational studies

|  | Item No. | | | Recommendation | Page  No. | | |
| --- | --- | --- | --- | --- | --- | --- | --- |
| **Title and abstract** | 1 | | | (*a*) Indicate the study’s design with a commonly used term in the title or the abstract | 3 | | |
|  |  |  |  | (*b*) Provide in the abstract an informative and balanced summary of what was done and what was found | 3 | | |
| Introduction | | | | | | | |
| Background/rationale | 2 | | | Explain the scientific background and rationale for the investigation being reported | 5-6 | | |
| Objectives | 3 | | | State specific objectives, including any prespecified hypotheses | 6 | | |
| Methods | | | | | | | |
| Study design | 4 | | | Present key elements of study design early in the paper | 7 | | |
| Setting | 5 | | | Describe the setting, locations, and relevant dates, including periods of recruitment, exposure, follow-up, and data collection | 7, 11 | | |
| Participants | 6 | | | (*a*) *Cohort study*—Give the eligibility criteria, and the sources and methods of selection of participants. Describe methods of follow-up  *Case-control study*—Give the eligibility criteria, and the sources and methods of case ascertainment and control selection. Give the rationale for the choice of cases and controls  *Cross-sectional study*—Give the eligibility criteria, and the sources and methods of selection of participants | 7, 15 | | |
|  |  |  |  | (*b*) *Cohort study*—For matched studies, give matching criteria and number of exposed and unexposed  *Case-control study*—For matched studies, give matching criteria and the number of controls per case |  | | |
| Variables | 7 | | | Clearly define all outcomes, exposures, predictors, potential confounders, and effect modifiers. Give diagnostic criteria, if applicable | 8 | | |
| Data sources/ measurement | 8* | | | For each variable of interest, give sources of data and details of methods of assessment (measurement). Describe comparability of assessment methods if there is more than one group | 8 | | |
| Bias | 9 | | | Describe any efforts to address potential sources of bias | 8 | | |
| Study size | 10 | | | Explain how the study size was arrived at | 7 | | |
| Quantitative variables | | 11 | Explain how quantitative variables were handled in the analyses. If applicable, describe which groupings were chosen and why | | 8-9 | | |
| Statistical methods | | 12 | (*a*) Describe all statistical methods, including those used to control for confounding | | 8-9 | | |
|  |  |  | (*b*) Describe any methods used to examine subgroups and interactions | | 8-9 | | |
|  |  |  | (*c*) Explain how missing data were addressed | |  | | |
|  |  |  | (*d*) *Cohort study*—If applicable, explain how loss to follow-up was addressed  *Case-control study*—If applicable, explain how matching of cases and controls was addressed  *Cross-sectional study*—If applicable, describe analytical methods taking account of sampling strategy | |  | | |
|  |  |  | (*e*) Describe any sensitivity analyses | | 8-9 | | |
| Results | | | | | | |  |
| Participants | | 13* | (a) Report numbers of individuals at each stage of study—eg numbers potentially eligible, examined for eligibility, confirmed eligible, included in the study, completing follow-up, and analysed | | 11 |  |  |
|  |  |  | (b) Give reasons for non-participation at each stage | |  |  |  |
|  |  |  | (c) Consider use of a flow diagram | |  |  |  |
| Descriptive data | | 14* | (a) Give characteristics of study participants (eg demographic, clinical, social) and information on exposures and potential confounders | | 11 |  |  |
|  |  |  | (b) Indicate number of participants with missing data for each variable of interest | |  |  |  |
|  |  |  | (c) *Cohort study*—Summarise follow-up time (eg, average and total amount) | | 11 |  |  |
| Outcome data | | 15* | *Cohort study*—Report numbers of outcome events or summary measures over time | | 14 |  |  |
|  |  |  | *Case-control study—*Report numbers in each exposure category, or summary measures of exposure | | 15-18 |  |  |
|  |  |  | *Cross-sectional study—*Report numbers of outcome events or summary measures | |  |  |  |
| Main results | | 16 | (*a*) Give unadjusted estimates and, if applicable, confounder-adjusted estimates and their precision (eg, 95% confidence interval). Make clear which confounders were adjusted for and why they were included | | 15-18 |  |  |
|  |  |  | (*b*) Report category boundaries when continuous variables were categorized | |  |  |  |
|  |  |  | (*c*) If relevant, consider translating estimates of relative risk into absolute risk for a meaningful time period | |  |  |  |
| Other analyses | | 17 | Report other analyses done—eg analyses of subgroups and interactions, and sensitivity analyses | | SI |  |  |
| Discussion | | | | | | |  |
| Key results | | 18 | Summarise key results with reference to study objectives | | 19 |  |  |
| Limitations | | 19 | Discuss limitations of the study, taking into account sources of potential bias or imprecision. Discuss both direction and magnitude of any potential bias | | 21 |  |  |
| Interpretation | | 20 | Give a cautious overall interpretation of results considering objectives, limitations, multiplicity of analyses, results from similar studies, and other relevant evidence | | 19-21 |  |  |
| Generalisability | | 21 | Discuss the generalisability (external validity) of the study results | | 21 |  |  |
| Other information | | |  | | | |  |
| Funding | | 22 | Give the source of funding and the role of the funders for the present study and, if applicable, for the original study on which the present article is based | | 2 |  |  |

*Give information separately for cases and controls in case-control studies and, if applicable, for exposed and unexposed groups in cohort and cross-sectional studies.
